# The Mitogenome of the Subarctic Octocoral *Alcyonium digitatum* Reveals a Putative tRNA^Pro^ Gene Nested within MutS

**DOI:** 10.3390/cimb46080479

**Published:** 2024-07-27

**Authors:** Alisa Heuchel, Åse Emblem, Tor Erik Jørgensen, Truls Moum, Steinar Daae Johansen

**Affiliations:** 1 Genomic Division, Faculty of Biosciences and Aquaculture, Nord University, 8049 Bodø, Norway; alisa.heuchel@polar.se (A.H.); tor.e.jorgensen@nord.no (T.E.J.); truls.g.moum@nord.no (T.M.); 2 Abisko Scientific Research Station, Swedish Polar Research Secretariat, SE-981 07 Abisko, Sweden; 3 Research Laboratory, Nordland Hospital Trust, 8005 Bodø, Norway

**Keywords:** Alcyoniidae, dual coding, mitochondrial genome, octocoral, phylogeny, tRNA

## Abstract

We sequenced and analyzed the complete mitogenome of a Norwegian isolate of the octocoral *Alcyonium digitatum* using the Ion Torrent sequencing technology. The 18,790 bp circular mitochondrial genome was found to harbor the same set of 17 genes, which encode 14 protein subunits, two structural ribosomal RNAs and one tRNA, as reported in other octocorals. In addition, we detected a new tRNA^Pro^-like gene sequence nested within the MutS protein coding region. This putative tRNA gene feature appears to be conserved among the octocorals but has not been reported previously. The *A. digitatum* mitogenome was also shown to harbor an optional gene (ORFA) that encodes a putative protein of 191 amino acids with unknown function. A mitogenome-based phylogenetic analysis, presented as a maximum likelihood tree, showed that *A. digitatum* clustered with high statistical confidence with two other *Alcyonium* species endemic to the Mediterranean Sea and the Southeast Pacific Ocean.

## 1. Introduction

Octocorallia (class Anthozoa) represents an important subclass of marine cnidarians that hosts the well-known orders Pennatulacea (sea pens) and Alcyonacea (soft corals and sea fans). The order Alcyonacea consists of about 2700 valid species organized into 30 families, mostly found in warm, shallow waters [1]. *Alcyonium digitatum* (dead man’s fingers), however, is a cold-water soft coral in the family Alcyoniidae. *A. digitatum* is confined to the North Atlantic Ocean and commonly present in Norwegian coastal waters [2].

Mitochondrial genomes (mitogenomes) in octocorals vary in size from approximately 18 to 20 kb and are highly economically organized [3,4,5]. Rearrangements are relatively common and both strands are gene coding. The circular mitogenome codes for the same set of 13 oxidative phosphorylation (OxPhos) proteins and two ribosomal RNAs (rRNAs) as most metazoans. However, only one tRNA gene has been recognized, indicating an extensive mitochondrial import of cytoplasmic tRNAs to support mitochondrial translation. The highly reduced tRNA gene repertoire also challenges the mitochondrial mRNA processing pattern, which is complex and clearly different from the tRNA punctuation pathway known, for example, in vertebrates [6,7].

A hallmark of octocoral mitogenomes is the presence of a MutS homolog gene (*mutS*). Bacterial MutS proteins are involved in DNA mismatch repair and recombination [8], and similar roles have been suggested for the mitochondrial homolog [9]. The mutS gene, along most mitochondrial protein-coding genes (PCGs) in octocorals, is under strong purifying selection [10,11] and encodes a multidomain protein of approximately 980 amino acids [12]. Interestingly, several distinct mRNA variants are generated during RNA processing and maturation, which involve alternative 5′ ends and at least seven different polyadenylation sites [7]. Why the mutS gene has become a compulsory feature in octocoral mitogenomes is currently not fully understood. Here, we report the complete mitogenome sequence from a subarctic specimen of *A. digitatum* (Nor-1). The analyses revealed the presence of a new tRNA gene feature nested within the MutS coding region, which may be conducive to the mutS gene retention among octocoral species.

## 2. Materials and Methods

### 2.1. DNA Extraction and Sequencing

We determined the complete mitogenome sequence from a subarctic specimen of *A. digitatum* (Nor-1) sampled at the coast of northern Norway (Bodø, Nordland County; 67°16′ N, 14°24′ E). Tissue sample from the body wall was preserved in absolute ethanol at −20 °C and subsequently stored in Nord University’s tissue collection at −80 °C. Total DNA was extracted as described by the supplier using the Epicentre^TM^ MasterPure^TM^ Complete DNA and RNA Purification Kit (Biosearch^TM^ Technologies, Hoddesdon, UK). DNA was subjected to whole-genome Ion Torrent PGM sequencing (Thermo Fisher Scientific, Waltham, MA, USA) using 316 v.2 chips as previously reported [13]. In short, sequencing libraries were constructed from DNA fragment sizes selected to be approximately 400 bp, and a total of 3.0 million reads were obtained from *A. digitatum*.

### 2.2. Data Analysis

The assembly was based on the MITObim script [14] and revealed a contig of 18,790 bp, which corresponded to the circular mitogenome. The maximum likelihood (ML) tree-building method in MEGA 11 was used to infer the molecular phylogeny, and the sequence alignment was model tested prior to tree construction [15]. The best-fit model was the general time reversible (GTR) with gamma-distributed (+G) rates across sites. The tree topology was evaluated using 2000 bootstrap replicates. An unambiguous sequence alignment of approximately 11,700 nucleotide positions representing all 13 concatenated OxPhos protein-coding genes (PCGs) was used to reconstruct the mitogenome phylogeny.

## 3. Results and Discussion

### 3.1. Canonical Mitochondrial Gene Features

**Figure 1:**
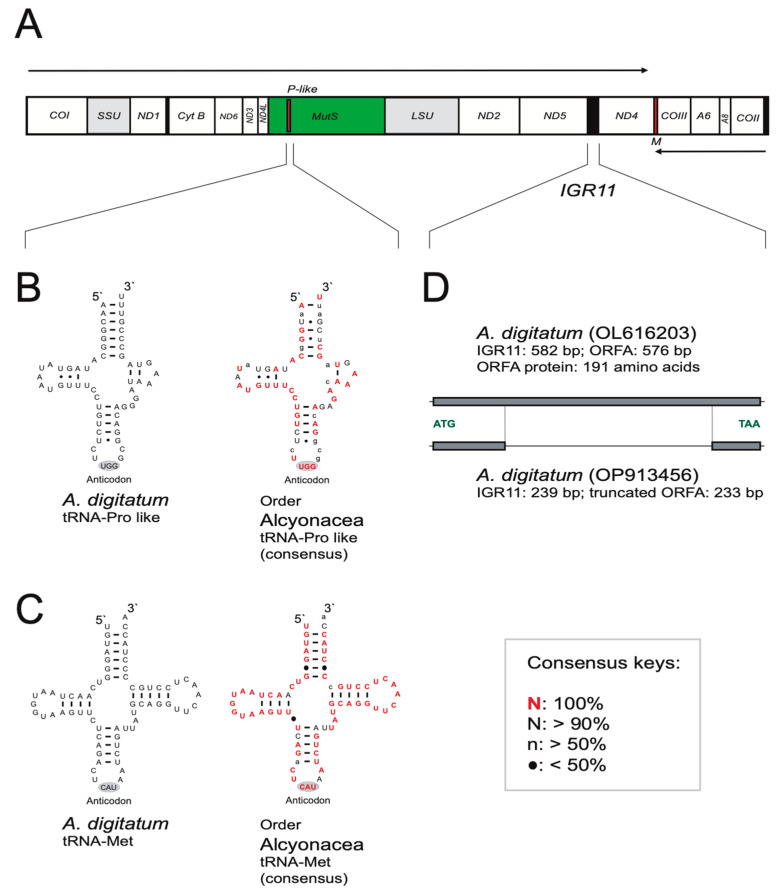
Mitogenome structural features of *Alcyonium digitatum*. (**A**) The circular mitogenome content and organization presented as a linear map. Horizontal arrows indicate the forward and reverse orientations. Gene annotations: COI to III, cytochrome c oxidase subunits I to III; ND1 to 6, NADH dehydrogenase subunits 1 to 6; ATP6 and 8, ATPase subunits 6 and 8; CytB, cytochrome b subunit; MutS, mitochondrial mutation suppressor homolog; ORFA, open reading frame A of unknown function; SSU and LSU rRNA, mitochondrial small and large subunit ribosomal RNAs; M, tRNA^Met^; P-like, tRNA^Pro^ -like. (**B**) Secondary structure of the tRNA^Pro^ -like RNA in *A. digitatum* (left) and a consensus structure for the order Alcyonacea based on one representative from each of 12 families (right): Alcyoniidae (OP913456), Isididae (EF672731), Briareidae (DQ640649), Acanthogorgiidae (GU047880), Clavulariidae (MT161608), Coralliidae (NC_015406), Nephtheidae (FJ372991), Plexauridae (HQ694727), Gorgoniidae (KY559408), Ellisellidae (KJ541509), Paragorgiidae (KF785800), and Primnoidae (KM015351). (**C**) Secondary structure of tRNA^Met^ from *A. digitatum* and a consensus structure for the order Alcyonacea for comparison, based on the same mitogenome sequences as above. (**D**) Extended view of IGR-11 harboring the ORFA gene coding a putative 191 amino acid protein in *A. digitatum* (OL616203) and a truncated protein in *A. digitatum* (OP913456).

The 18,790 bp mitogenome was sequenced with high coverage (159 times average coverage), has a low GC content (37%), a high gene density (97%), and contains the same set of 13 OxPhos PCGs as most metazoan mitochondrial genomes, as well as the *mutS* (Figure 1A; Table S1). The mitogenome also encodes small subunit (SSU) and large subunit (LSU) rRNAs and tRNA^Met^. No introns are present, which is a distinguishing feature of the subclass Octocorallia compared to the related Anthozoan subclass Hexacorallia [16]. In *A. digitatum,* most mitochondrial genes are encoded by the same strand, but with five exceptions (Figure 1A). Thus, the gene order and organization appear similar to that of most Alcyonacea mitochondrial genomes [3,4,5].

### 3.2. Non-Canonical Mitochondrial Gene Features

We noted two additional and previously unannotated putative genes in the *A. digitatum* mitogenome; a putative tRNA^Pro^ gene and an ORFA gene encoding a 191 aa protein of unknown function.

#### 3.2.1. Putative tRNA^Pro^ Gene

A tRNA-like gene was found nested within the MutS coding region (Figure 1A). The derived tRNA structure resembles that of tRNA^Pro^ and appears to be highly conserved within the order Alcyonacea (Figure 1B; Figure S1; Table S2), corresponding to an evolutionary time of approximately 500 million years [17]. Furthermore, its folding pattern and level of conservation is similar to that of tRNA^Met^ (Figure 1C). Two closely related Scleractinian hexacoral species, *Porites rus* and *Porites lutea*, are also reported to encode a putative tRNA^Pro^ gene in their mitogenomes [18,19]. Interestingly, the putative tRNA gene is embedded in a mitochondrial non-canonical homing endonuclease gene within the COI group I intron. The dual coding functions of DNA segments are also known from other hexacoral mitogenomes. Specifically, the COI group I introns in several sea anemones and mushroom corals species encode both a catalytic RNA and an endonuclease protein from the same sequence [20,21,22]. Another well-characterized example is the vertebrate mitochondrial-derived peptides encoded within the SSU rRNA and LSU rRNA genes [23,24,25]. The octocoral *mutS* evolution and function appear complex and obscure [12], including different mRNAs with alternative 5′ and 3′ ends [7]. Thus, it appears plausible that *mutS* may have a dual coding capacity of protein and structural RNA, which would also be conducive to its retention among octocoral species, but this feature needs to be further investigated in a functional context.

#### 3.2.2. ORFA Gene

The intergenic region 11 (IGR-11) appeared variable in size in *Alcyonium*. While IGR-11 in one isolate of *A. digitatum* (OL616203) was 582 bp, the Nor-1 (OP913456) isolate was found to be 239 bp (Figure 1D; Figure S2). Furthermore, the 97 bp IGR-11 of *A. haddoni* (NC_061993) is identical to that of *A. acaule* (NC_061273) but different to that of *A. digitatum*. A closer inspection of the 582 bp region in *A. digitatum* revealed an open reading frame (named ORFA) encoding a putative and currently unknown protein of 191 amino acids (Figure 1D). The corresponding 239 bp region in Nor-1 represented a truncated version of ORFA. A BLAST search of the 582 bp IGR-11 identified related but truncated sequences in several octocoral mitogenomes of genera such as *Eunicella* (OL616234, KY559407, KY559408, and MW588805), *Paraminabea* (OL616255), *Cladiella* (OL616222), *Paramuricea* (OL616256 and LT576168), *Dendronephthya* (OL616226), *Echinogorgia* (KY230385 and HQ694727) and *Muricea* (LT174652 and LT174653). This suggests a recent gain and loss of gene sequences in *A. digitatum* and several other octocorals, a feature resembling the dynamic mitogenome structure reported in hexacorals [16,20].

### 3.3. Phylogenetic Considerations

**Figure 2:**
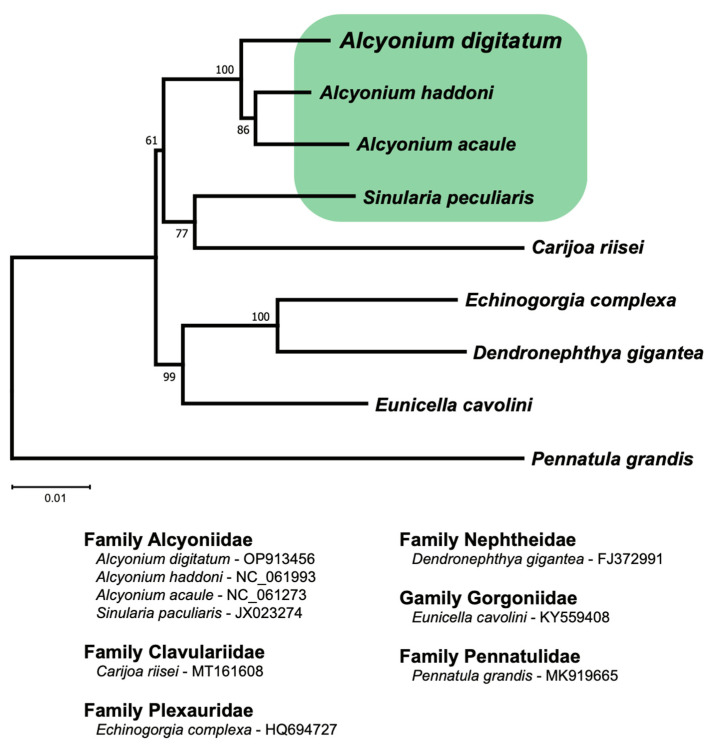
Molecular phylogeny of *Alcyonium digitatum* and some related octocoral species based on concatemeric mitochondrial protein-coding sequences (11,700 positions) and maximum likelihood analysis (2000 bootstrap replicates). Bootstrap support values are indicated at the branch nodes. The sea pen *Pennatula grandis* was used as the outgroup. Specimens from family Alcyoniidae are boxed. The scale bar indicates the fraction of substitutions per site.

An alignment of 11,700 nucleotide positions representing all 13 concatenated OxPhos PCGs was used to reconstruct the mitogenome phylogeny of nine species representing six families of octocorals. The sea pen *Pennatula grandis* (order Pennatulacea) was used as the outgroup in the analysis. SSU and LSU rRNA genes and MutS gene sequences were excluded from the alignment due to frequent indels among the octocoral mitochondrial genomes assessed. The resulting phylogenetic tree (Figure 2) showed that the *A. digitatum* isolates cluster with high confidence (bootstrap value 100%) to the congeneric *A. acaule* (endemic to the Mediterranean Sea) and *A. haddoni* (endemic to the Southeast Pacific Ocean).

## 4. Conclusions

We report new structural features in the mitogenome sequence of *A. digitatum* with general relevance to octocorals that include a putative tRNA gene and a protein-coding gene with an unknown function. A biological role of the putative tRNA^Pro^ is currently not known, but we speculate that it could act as a regular tRNA in mitochondrial translation. The MutS coding region in *A. digitatum* contains as much as 44 proline codons, and an internal tRNA^Pro^ gene may thus compensate for an increased demand of tRNA^Pro^ during translation. Alternatively, the putative tRNA^Pro^ structure may be involved in RNA processing and the maturation of the *mutS* mRNA variants by recruiting mitochondrial RNaseP.

## Data Availability

The DNA raw-read sequencing data are assigned the accession number PRJNA910106 in the NCBI’s Sequence Read Archive (SRA). The complete mitogenome sequence of *Alcyonium digitatum* (Nor-1) is assigned the accession number OP913456.

## Supplementary Materials

The following supporting information can be downloaded at: https://www.mdpi.com/article/10.3390/cimb46080479/s1, Figure S1: Secondary structure of putative tRNA^Pro^ from representative mitogenomes of twelve families within the order Alcyonacea; Figure S2: Sequential features of the intergenic region 11 (IGR-11) in *A. digitatum*; Table S1: Annotation of *A. digitatum* (Nor-1) complete mitochondrial genome; Table S2: List of mitogenome sequences (order Alcyonacea) included in the tRNA consensus structure analyses.
