# Supplementary material for: The Mitogenome of the Subarctic Octocoral Alcyonium digitatum Reveals a Putative tRNAPro Gene Nested within MutS
Source: Curr Issues Mol Biol. 2024 Jul 27;46(8):8104–10. doi: 10.3390/cimb46080479 (PMC11353228; doi:10.3390/cimb46080479)
Supplement: Supplementary file 1 [file cimb-46-00479-s001.zip › cimb-3115405-supplementary.pdf]

Supplementary Materials

The mitogenome of the subarctic octocoral *Alcyonium digitatum* reveals a putative $tRNA^{Pro}$ gene nested within *mutS*

Alisa Heuchel, Åse Emblem, Tor Erik Jørgensen, Truls Moum and Steinar Daae Johansen

Figure S1. Secondary structure of putative $tRNA^{Pro}$ from representative mitogenomes of twelve families within the order Alcyonacea.

Figure S2. Sequential features of the intergenic region 11 (IGR-11) in *A. digitatum*. Sequence comparison of IGR-11 in OP913456 (OP) and OL616203 (OL) and derived amino acid sequence of ORFA protein in OL616203.

Table S1. Annotation of *A. digitatum* (Nor-1) complete mitochondrial genome.

Table S2. List of mitogenome sequences (order Alcyonacea) included in the putative tRNA consensus structure analyses.

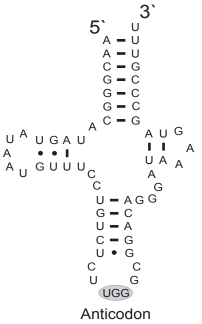

Alcyonium digitatum
Putative tRNA-Pro
Family Alcyoniidae
OP913456

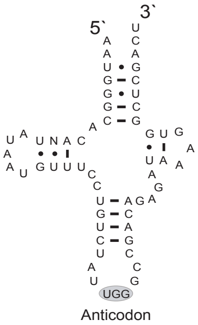

Acanella eburnea
Putative tRNA-Pro
Family Isididae
EF672731

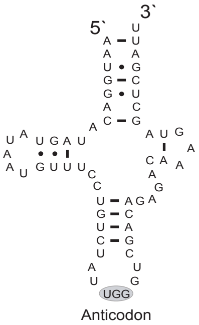

Briareum asbestinum
Putative tRNA-Pro
Family Briareidae
DQ640649

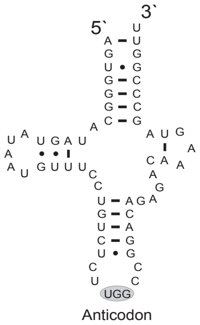

Calicogorgia granulosa
Putative tRNA-Pro
Family Acanthogorgiidae
GU047880

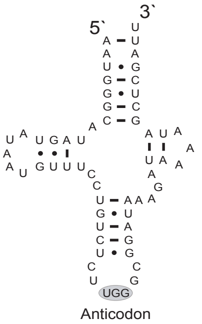

Carijoa riisei
Putative tRNA-Pro
Family Clavulariidae
MT161608

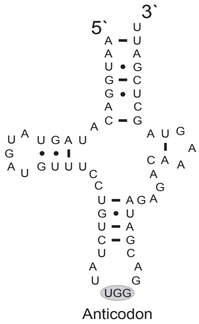

Corallium konojoi
Putative tRNA-Pro
Family Coralliidae
NC_015406

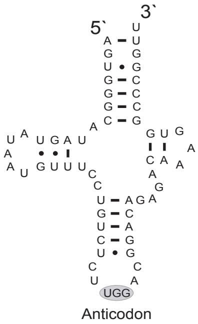

Dendronephthya gigantea
Putative tRNA-Pro
Family Nephtheidae
FJ372991

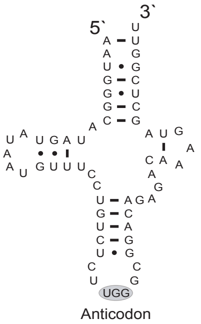

Echinogorgia complexa
Putative tRNA-Pro
Family Plexauridae
HQ694727

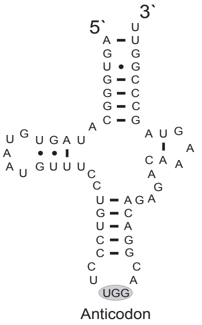

Eunicella cavolini
Putative tRNA-Pro
Family Gorgoniidae
KY559408

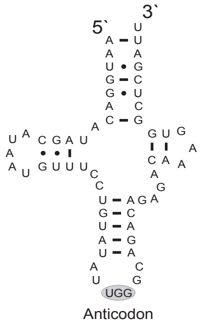

Junceella fragilis
Putative tRNA-Pro
Family Ellisellidae
KJ541509

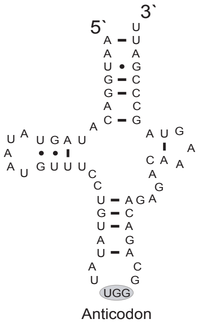

Paragorgia coralloides
Putative tRNA-Pro
Family Paragorgiidae
KF785800

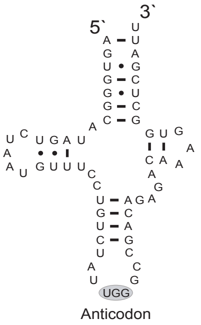

Narella hawaiiensis
Putative tRNA-Pro
Family Primnoidae
KM015351

Figure S1

Figure S2.

Sequential features of the intergenic region 11 (IGR-11) in *A. digitatum*.

Sequence comparison of IGR-11 in OP913456 (OP) and OL616203 (OL), and derived amino acid sequence of ORFA protein in OL616203.

```
               M  K  K  T  R  N  F  Y  I  T  Y  C  R  W  I  L  A  F  L  L  C  H  P  I
OL          ACGTATGAAGAAAACCAGAAATTTTTATATAACTTATTGTAGATGAATATTAGCTTTTTTATTGTGCCACCCTAT
OP          ACGTATGAAGAAAACCAGAAATTTTTATATAACTTATTGTAGATGAATATTAGCTTTTTTATTGTGCCACCCTAT

             I  F  R  V  I  G  V  V  I  C  V  L  M  F  I  V  E  I  V  W  S  G  P  V  Y
OL          AATATTTAGAGTAATAGGTGTAGTTATATGTGTGTTAATGTTTATAGTTGAGATTGTTTGGTCTGGCCCCGTATA
OP          AATATTTAGAGTAATAGGTGTAGTTATATGTGTGTTAATGTTTATAGTTG-------------------------

             A  S  G  P  I  E  S  F  I  G  S  Y  L  A  Y  D  P  Y  L  A  Y  G  P  Y  L
OL          CGCCTCCGGGCCAATAGAAAGCTTTATTGGCTCTTACTTGGCATATGACCCTTACTTGGCATATGGCCCTTACTT

             A  Y  G  P  L  D  P  L  P  P  I  I  G  N  V  S  I  L  S  P  P  P  L  P  F
OL          GGCATATGGCCCTTTGGATCCACTACCTCCTATAATAGGTAATGTCAGTATACTATCGCCCCCTCCACTTCCTTT

             N  Y  S  S  V  L  T  L  T  L  M  Y  E  P  M  E  I  E  Y  G  L  V  N  T  L
OL          TAACTATTCCTCTGTATTGACTCTGACTCTAATGTACGAGCCTATGGAGATTGAATATGGGCTAGTTAACACTTT

             N  N  P  D  F  Y  I  G  Q  H  I  E  G  P  I  V  S  M  E  T  V  I  E  Q  G
OL          AAATAATCCAGATTTTTACATAGGCCAACATATAGAGGGGCCAATAGTAAGCATGGAAACAGTCATTGAACAAGG

             W  V  C  T  T  I  N  N  D  T  S  Y  V  F  S  R  H  N  G  R  L  M  R  E  Y
OL          CTGGGTCTGTACTACTATCAATAATGATACATCGTATGTATTTAGTCGGCATAATGGGCGGCTCATGCGGGAGTA
OP          ------------------CAATAATGATACATCGTATGTATTTAGTCGGCATAATGGGCGGCTCATGCGGGAGTA

             T  P  A  E  F  A  A  S  N  W  V  A  D  A  E  V  L  *               191 aa
OL          TACCCCCGCCGAGTTTGCGGCGAGCAACTGGGTAGCGGACGCTGAGGTTCTATAATT               582 bp
OP          TACCCCCGCCGAGTTTGCGGCGAGCAACTGGGTAGCGGACGCTGAGGTTCTATAATT               239 bp
```

Table S1.

Annotation of *A. digitatum* (Nor-1) complete mitochondrial genome.

**Gene/ region** [1]	**Strand** [2]	**Position** [3]	**Size (bp)**	**Size (aa)**	**Start/stop** [4]
COI	F	1 – 1582	1582	527	ATG/Taa
IGR-1		-	0		
SSU rRNA	F	1583 – 2624	1042		
IGR-2		2625 – 2666	42		
ND1	F	2667 – 3635	969	322	ATG/TAG
IGR-3		3636 – 3710	75		
CytB	F	3711 - 4853	1143	380	ATG/TAA
IGR-4		4854 – 4880	27		
ND6	F	4881 - 5438	558	185	ATG/TAG
IGR-5		5439 – 5493	55		
ND3	F	5494 - 5847	354	117	ATG/TAG
IGR-6		5848 – 5865	18		
ND4L	F	5866 - 6159	294	97	ATG/TAA
IGS-7		6160 – 6172	13		
MutS	F	6173 – 9115	2943	980	ATG/TAA
tRNA^{Pro} (P)-like	F	6680 – 6738	59		
IGR-8		9116 – 9124	9		
LSU rRNA	F	9125 – 11061	1937		
IGR-9		11062 – 11087	26		
ND2	F	11088 - 12449	1362	453	ATG/TAG
IGR-10		-	- 13		
ND5	F	12437 - 14254	1818	605	ATG/TAG
IGR-11		14255 – 14493	239		
Truncated ORFA	F	14259 – 14491	233		
ND4	F	14494 - 15942	1449	482	ATG/TAA
IGR-12		15943 – 16068	56		
tRNA^{Met} (M)	R	16069 – 15999	71		
IGR-13		16070 – 16106	37		
COIII	R	16892 - 16107	786	261	ATG/TAG
IGR-14		16893 – 16954	62		
ATP6	R	17662 - 16955	708	235	ATG/TAA
IGR-15		17663 – 17685	23		
ATP8	R	17901 - 17686	216	71	ATG/TAG
IGR-16		17902 – 17922	21		
COII	R	18684 - 17923	762	253	ATG/TAG
IGR-17		18685 – 18790	106		

Notes: [1] Abbreviations of mitochondrial genes and intergenic regions (IGR-1 to 17). COI to III, cytochrome c oxidase subunits I to III; ND1 to 6, NADH dehydrogenase subunits 1 to 6; ATP6 and 8, ATPase subunits 6 and 8; CytB, cytochrome b subunit; MutS, mitochondrial mutation suppressor homolog; ORFA, open reading frame A of unknown function; SSU and LSU rRNA, mitochondrial small and large subunit ribosomal RNA; [2] F, forward strand; R, reverse strand. [3] Positions are according to the accession number OL913456. [4] Start and stop codons of open reading frames. Stop codon of COI is proposed generated by posttranscriptional polyadenylation.

Table S2.
List of mitogenome sequences (order Alcyonacea) included in the tRNA consensus structure analyses.

Family	Species	Accession number
Alcyoniidae	*Alcyonium digitatum*	OP913456
Isididae	*Acanella eburnea*	EF672731
Briareidae	*Briareum asbestinum*	DQ640649
Acanthogorgiidae	*Calicogorgia granulosa*	GU047880
Clavulariidae	*Carijoa riisei*	MT161608
Coralliidae	*Corallium konojoi*	NC_015406
Nephtheidae	*Dendronephthya gigantea*	FJ372991
Plexauridae	*Echinogorgia complexa*	HQ694727
Gorgoniidae	*Eunicella cavolini*	KY559408
Ellisellidae	*Junceella fragilis*	KJ541509
Paragorgiidae	*Paragorgia coralloides*	KF785800
Primnoidae	*Narella hawaiiensis*	KM015351
